# HC-Pro silencing suppressor significantly alters the gene expression profile in tobacco leaves and flowers

**DOI:** 10.1186/1471-2229-11-68

**Published:** 2011-04-20

**Authors:** Arto J Soitamo, Balaji Jada, Kirsi Lehto

**Affiliations:** 1 Department of Biochemistry and Food Chemistry, Molecular Plant Biology, University of Turku, Vesilinnantie 5, Turku, 20014, Finland

## Abstract

**Background:**

RNA silencing is used in plants as a major defence mechanism against invasive nucleic acids, such as viruses. Accordingly, plant viruses have evolved to produce counter defensive RNA-silencing suppressors (RSSs). These factors interfere in various ways with the RNA silencing machinery in cells, and thereby disturb the microRNA (miRNA) mediated endogene regulation and induce developmental and morphological changes in plants. In this study we have explored these effects using previously characterized transgenic tobacco plants which constitutively express (under CaMV 35S promoter) the helper component-proteinase (HC-Pro) derived from a potyviral genome. The transcript levels of leaves and flowers of these plants were analysed using microarray techniques (Tobacco 4 × 44 k, Agilent).

**Results:**

Over expression of HC-Pro RSS induced clear phenotypic changes both in growth rate and in leaf and flower morphology of the tobacco plants. The expression of 748 and 332 genes was significantly changed in the leaves and flowers, respectively, in the HC-Pro expressing transgenic plants. Interestingly, these transcriptome alterations in the HC-Pro expressing tobacco plants were similar as those previously detected in plants infected with ssRNA-viruses. Particularly, many defense-related and hormone-responsive genes (e.g. ethylene responsive transcription factor 1, ERF1) were differentially regulated in these plants. Also the expression of several stress-related genes, and genes related to cell wall modifications, protein processing, transcriptional regulation and photosynthesis were strongly altered. Moreover, genes regulating circadian cycle and flowering time were significantly altered, which may have induced a late flowering phenotype in HC-Pro expressing plants. The results also suggest that photosynthetic oxygen evolution, sugar metabolism and energy levels were significantly changed in these transgenic plants. Transcript levels of S-adenosyl-L-methionine (SAM) were also decreased in these plants, apparently leading to decreased transmethylation capacity. The proteome analysis using 2D-PAGE indicated significantly altered proteome profile, which may have been both due to altered transcript levels, decreased translation, and increased proteosomal/protease activity.

**Conclusion:**

Expression of the HC-Pro RSS mimics transcriptional changes previously shown to occur in plants infected with intact viruses (e.g. *Tobacco etch virus*, TEV). The results indicate that the HC-Pro RSS contributes a significant part of virus-plant interactions by changing the levels of multiple cellular RNAs and proteins.

## Background

Plant virus infections cause a large variety of different disease symptoms in susceptible plants. Viruses invade and utilize the central biosynthetic routes of the host cells, but plants have evolved specific means to resist virus attacks. RNA silencing is one of the main adaptive defence mechanism against transposons, transgenes and also pathogenic nucleic acids i.e. viruses [1-3]. During viral RNA replication in plants, the viral ssRNA molecules produce dsRNA structures, which are processed by Dicer-like ribonucleases (DCL; an RNAse III-like enzyme) into small interfering RNAs (siRNAs). These assemble with argonaute (AGO) protein(s) to form the RNA-induced silencing complexes (RISC) that are able to specifically cleave RNAs sharing sequence identity with the original viral RNA (PTGS, post-transcriptional gene silencing) [4]. To counteract this host defence mechanism, viruses encode for specific RSSs. These counteract the degradation of viral RNA, but they also interfere with plants own small RNA (smRNA) biosynthesis and silencing-mediated gene regulation. It has been shown that the virus symptoms are induced at least to some extent by these factors, and that severe (symptom-like) developmental defects can be caused in vegetative and reproductive organs by their transgenic expression [5-13].

Proteinase1/Helper component-proteinase (P1/HC-Pro) encoded by the 5' proximal region of the TEV was one of the first RSSs characterized [14]. Since then, features of the HC-Pro RSS of different potyviruses have been characterized in detail in several papers [5,6,11,13,15-18]. They have been shown to affect differently the accumulation of various miRNA molecules and miRNA target transcripts [5,6,11]. Both miRNA processing and function are impaired in transgenic P1/HC-Pro expressing lines, and consequently both the miRNA/ miRNA* processing intermediates and the miRNA target messages accumulate in these transgenic plants. More recently, it has been shown that the P1/HC-Pro directly binds and sequesters miRNA/miRNA* molecules [16]. It has been also shown that HC-Pro interacts with the 26S proteasomes [19] and inhibits their RNA endonuclease activity [20]. The plant proteasomes function as an anti-viral defence system by degrading virus RNAs, and potyviral HC-Pro counteracts also this anti-viral defence system by decreasing their endonuclease activity [20].

Most of the previous studies of the HC-Pro RSS have been performed using *Arabidopsis thaliana *as a model plant. Transgenic tobacco plants (a natural host of *Potato Virus Y*, PVY) which constitutively express PVY-derived HC-Pro, have been previously produced and characterized in our laboratory [10]. Here we have analysed by microarray techniques (Tobacco 4 × 44 k, Agilent) the transcript profiles of the leaves and flowers of these tobacco plants, and compared them to the previously published transcriptome analysis of virus-infected *A. thaliana *[15,21-26]. Array results indicated significant transcriptional changes both in the leaf and flower samples, especially in genes encoding proteins involved in plants defence, as well as in genes related to stress response, circadian and flowering time responses and energy metabolism. Most of these changes are similar with changes reported in the plants infected with intact RNA viruses, e.g. TEV and *Cucumber mosaic virus *strain Y (CMV-Y).

## Results

### Experimental design and differential gene expression

**Figure 1:**
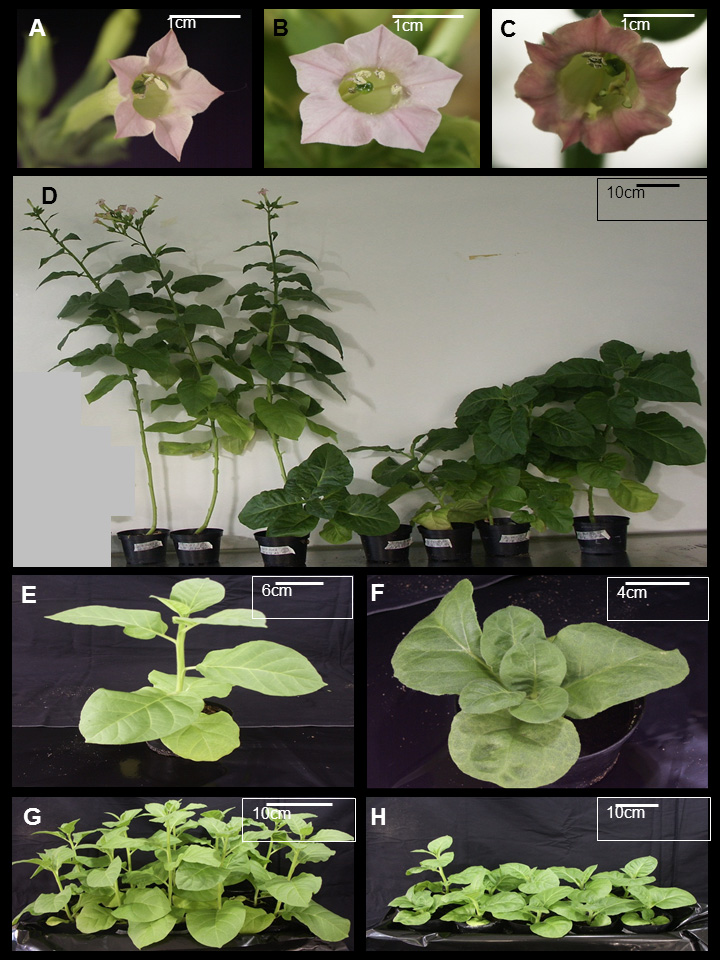
**Phenotypes observed in *Nicotiana tabacum *plants expressing *HC-Pro *transgene**. A typical morphology of flowers is indicated in the upper part of the figure (**A-C**). A wild type tobacco flower is presented in **A**, a vector control flower (pBIN61) in **B **and a transgenic HC-Pro expressing flower in **C**. Phenotypes of two wild type tobacco plants at the flowering state (on the left) and one vector control plant (pBIN61, in between of these wild type plants) and four transgenic HC-Pro expressing plants are presented in **D**. One representative of one-month old wild type tobacco plant (**E**) and one transgenic HC-Pro expressing plant (**F**) demonstrating differences in growth and leaf morphology. A growing pattern of 10 one-month old wild type tobacco plants (**G) **and 10 transgenic HC-Pro expressing plants (**H**) are presented at the bottom of the figure

The transgenic tobacco line expressing the *HC-Pro *gene of PVY strain N under constitutive expression of CaMV 35S promoter [10] was used in this study. Wild type tobacco (wt) plants and plants containing empty transformation vector (pBIN61) were used as controls for the transgenic line [10]. No phenotypic differences were detected between these two types of control plants (Figure 1A, 1B and 1D).

The expression of HC-Pro RSS in tobacco plants caused clear phenotypic changes in leaves, stems and flowers as earlier described [10]. The growth of transgenic HC-Pro expressing plant was clearly retarded, and the appearance of the plants varied from short stems to almost a bushy like appearance (Figure 1D). Also the flowering time was clearly delayed, as the transgenic plants typically flowered two to three months later than the wild type plants. The morphology of the flower was variable, but it often differed from the wild type. The petals were often fused together and the color of the petals was changed from pink to pale pink or variegated. The anther filaments were often converted to extra petals and sometimes they were divided (Figure 1C). The transgenic plants produced only small amount of viable seeds.

The expression level of *HC-Pro *transgene varied in tobacco plants and affected the phenotype of these transgenic plants; the higher HC-Pro expression levels the more severe developmental defects [10]. Out of ten plants, three plants were chosen for the microarray analysis based on typical, average phenotype of HC-Pro plants (see Figure 1) and on average transgene HC-Pro expression (Additional file 1).

The microarray was performed according to Agilent's standard protocols and quality controls for total RNA, and for cDNA labeling (see Methods). After data normalization of leaf and flower samples, statistical parameters for genes were calculated. Statistical differences between the two types of controls (wt and pBIN61) and HC-Pro transgenic leaf and flower samples were tested by using Student's t-test (p < 0.05). It turned out that the two types of control plants had a very similar expression pattern, with only a few genes being differentially expressed between them (Additional files 2 and 3). Therefore both control samples could be used together to make a total of six biological control replicates. Finally, the six normalized gene expression intensity values of control samples were compared against three normalized intensity values of the HC-Pro expressing transgenic plants to detect whether gene expression values would differ significantly (p < 0.05) from each other. A two-fold cut of value for up- or down- regulated genes were selected. Based on these comparisons 368 genes were found up-regulated and 380 genes down-regulated in leaves of the HC-Pro expressing plants, making together 748 differently expressed genes in the leaves. However, only 121 genes were up-regulated and 211 genes down-regulated in the HC-Pro expressing flower samples (Table 1).

**Table 1:** An overview of microarray results demonstrating differentially expressed transcripts in leaves and flowers in HC-Pro expressing plants

Functional characterization	HC-Pro leaf	HC-Pro leaf	HC-Pro flower	HC-Pro flower
Expression of genes	(UP)	(DOWN)	(UP)	(DOWN)
Defence related	7	17	21	12
ROI related	12	12	2	6
Kinases and phosphatases	10	20	1	12
Transcriptional regulators	21	28	9	7
Protein degradation and proteases	14	7	9	4
Lipases and hydrolases	10	8	0	4
Transporters	5	20	2	24
HSPs	7	7	1	0
Signalling	7	2	2	3
Cell wall related	16	11	9	29
Stress related	28	37	8	7
Protein synthesis related	9	4	1	1
Photosynthesis related	31	42	3	15
RNA binding	16	2	3	1
Interesting miscellaneous	120	101	21	65
Unknown function	55	62	29	21
Total	368	380	121	211

Table represents functional characterization of genes whose expression was up- or down- regulated more than two-fold. Statistical significance was tested by using Student's-test (p < 0.05).

The microarray results were verified by reverse transcription-quantitative PCR (RT-qPCR) of some significantly up- and down-regulated genes both in the leaf and flower samples (Table 2). Similar expression data was obtained for these selected genes using both these methods.

**Table 2:** Verification of microarray results using RT-qPCR

Leaf (up-regulated transcripts)		Microarray	RT-qPCR	
EST/mRNA	Description	Fold	Fold	s.e
EH620344	*Arabidopsis thaliana *FKF1 (FLAVIN-BINDING, KELCH REPEAT, F BOX 1)	12.45	18.36	2.97
EH615198	*Nicotiana tabacum *nictaba (NT1) mRNA Jasmonic acid methyl ester and ethylene-induced mRNA	6.41	10.1	2.90
FG156808	*Nicotiana tabacum *1-D-deoxyxylulose 5-phosphate synthase (DXS) mRNA	3.61	3.30	0.27
**Leaf (down-regulated transcript)**				
AY741503	*Nicotiana tabacum *S-Adenosyl- L-methionine methyl transferase mRNA (SAMT) (p = 0.067)	0.44	0.35	0.11
**Leaf (non-regulated transcripts)**				
EB450395	*Arabidopsis thaliana *ARPC3 (actin-related protein C3)	1.09	1.00	0.00
X67159	*Nicotiana tabacum *pectate lyase mRNA	0.98	1.01	0.01
**Flower (up-regulated transcripts)**				
EB438380	*Solanum lycopersicum *Trypsin and protease inhibitor, mRNA	2.86	3.50	0.94
EB683763	*Nicotiana tabacum *mRNA for P-rich protein NtEIG-C29	2.03	2.10	0.28
FG157361	*Nicotiana tabacum *mRNA for RAV	2.14	1.60	0.19
**Flower (down-regulated transcript)**				
AY772945	*Nicotiana tabacum *pectin methylesterase mRNA	0.36	0.19	0.16

Some clearly up- or down-regulated genes of leaf and flower samples were tested. Statistical significance was tested using Student's t-test (p < 0.05).

Fold change is indicated as a ratio of HC-Pro/WT calculated from normalized median intensity values (n = 3). Standard error of mean (s.e.) is also calculated for RT-qPCR values.

The construction of the microarray probes has been based mostly on tobacco EST, cDNA and mRNA sequences, and it was necessary to verify the gene names provided by Agilent. Thus, the genes that were found to be significantly up- or down- regulated was re-annotated using the BLAST program (NCBI). Additional information about the putative gene functions was obtained from recently sequenced tomato and potato genomes, as compared to the previous annotation solely based on *A. thaliana *genomic information. A summary of manually re-annotated and functionally characterized genes is presented in Table 1. Functional characterization was based on similar categorization presented by Marathe et al. [23].

### *HC-Pro *transgene causes virus infection-like changes in gene expression and induces defence-related genes

The microarray results (Table 1) clearly demonstrated that expression of HC-Pro in transgenic plants mimicked the effects of virus infections at the transcriptional level [15,21-26], as similar groups of genes were modulated in these plants as in *Arabidopsis *model plants infected by TEV [15] or CMV-Y [23].

Many defense and stress related genes were induced in both leaves and flowers of the HC-Pro expressing transgenic plants (Table 3). Many of these genes are regulated either by ethylene or jasmonic acid regulated pathways and can be induced by external treatment of these plant hormones. They can also be induced in transgenic *Arabidopsis *plants by over expression of the ethylene response transcription factor 1 (ERF1), which integrates signals from ethylene and jasmonic acid pathways in plant defense responses [27]. The expression of the ERF1 mRNA was up-regulated more than five times in leaves, and more than two times in flowers of the HC-Pro expressing transgenic tobacco plants (Table 4). In addition, ethylene response transcription factor 4 (ERF4), a negative regulator of jasmonic acid-responsive defence related genes [28] was clearly down-regulated in these plants. Accordingly, several jasmonic acid, ethylene or salicylic acid responsive transcription factors, like WIZZ (a JA-induced WRKY protein), Jasmonic acid 2 (a NAC transcription factor) and ethylene responsive transcription factor 3 (ERF3) were over expressed in HC-Pro expressing transgenic plants (Table 4). In flowers, the ERF1 transcription factor-induced genes include many genes encoding Avr9/Cf9 rapidly elicited (ACRE) proteins. Further annotation of these *ACRE *genes revealed that they were involved in both defense and stress responses, encoding for example proline rich proteins (e.g. cereal-type alpha-amylase inhibitors), lipid transfer proteins, seed storage proteins, late embryogenesis proteins (LEA), Avirulence-like protein 1, as well as AP2-type transcription factors (ACRE111B) (Additional files 4, 5 and 6).

**Table 3:** Up- or down-regulation of transcripts in HC-Pro expressing plants

Defense related transcripts Leaf			Stress related transcripts Leaf		
EST/mRNA	Fold	Description	EST/mRNA	Fold	Description
EH615198	6.4	*Nicotiana tabacum *nictaba mRNA (NT1)	TA14956_4097	3.8	*Tamarix *Putative stress-responsive protein
FG636567	3.8	*Nicotiana tabacum *mRNA for P-rich protein NtEIG-C29	FG156808	3.6	*Nicotiana tabacum *1-D-deoxyxylulose 5-phosphate synthase mRNA (DXS1)
EB433973	2.8	Parsley PcPR1-3 mRNA for pathogenesis-related protein type B	CV016057	2.9	*Arabidopsis thaliana *cold-regulated 413-plasma membrane 2 mRNA COR413-PM2
S44869	2.4	*Nicotiana tabacum *Endochitinase A precursor	EB441160	2.9	*Solanum lycopersicum *dxs2 gene for 1-deoxy-D-xylulose 5-phosphate synthase
X12739	2.1	*Nicotiana tabacum *Pathogenesis-related protein R major form precursor	EB435759	2.8	*Ipomoea nil *In04 mRNA for caffeoyl-CoA O-methyltransferase
TA14009_4097	0.5	*Nicotiana tabacum *Chitinase 134	TA12600_4097	2.8	*Solanum tuberosum *Low temperature and salt responsive protein
EB425556	0.5	*Arabidopsis thaliana *beta-1,3-glucanase-related mRNA	EB451519	2.8	*Solanum lycopersicum *geranylgeranyl pyrophosphate synthase 1 (GGPS1)
EH624302	0.5	*Arabidopsis thaliana *callose synthase 1 mRNA CALS1	EB445705	2.7	*Vitis vinifera *RD22-like protein mRNA
**Defense related transcripts Flower**			DV161729	2.1	*Arabidopsis thaliana *snf1-related protein kinase 2.2, SNRK2.2
FG167555	3.8	*Nicotiana tabacum *Avr9/Cf-9 rapidly elicited protein 111B (ACRE111B) AP2-DOMAIN	FG633784	2.0	*Solanum lycopersicum *anthocyanin acyltransferase mRNA, Jasmonic acid inducible
AB041516	3.5	*Nicotiana tabacum *P-rich protein EIG-I30	EB680165	0.3	*Arabidopsis thaliana *SIP3 (SOS3-INTERACTING PROTEIN 3)
TA14524_4097	3.0	*Nicotiana tabacum *Avr9/Cf-9 rapidly elicited protein 65 (ACRE65) mRNA,	EB433693	0.4	*Arabidopsis thaliana *AFP1 (ABI FIVE BINDING PROTEIN)
FG640154	2.6	*Nicotiana tabacum *mRNA for basic pathogenesis-related protein Thaumatin	TA14058_4097	0.4	*Ipomoea nil *CHS-D mRNA for chalcone synthase
FG635113	2.5	*Nicotiana tabacum *Avr9/Cf-9 rapidly elicited protein 20 (ACRE20) mRNA; EF-hand calcium binding protein	EB439278	0.4	*Nicotiana tabacum *NtERD10B mRNA for dehydrin
TA13004_4097	2.2	*Nicotiana tabacum *mRNA for hin1 gene: Harpin inducing protein	EB437158	0.5	*Ricinus communis *leuco-anthocyanidin dioxygenase mRNA
TA15227_4097	2.1	*Nicotiana tabacum *Avr9/Cf-9 rapidly elicited protein 76; NDR1/HIN1	DW003496	0.5	*Ricinus communis *Salt-tolerance protein
AB127582	2.1	*Nicotiana tabacum *Harpin inducing protein 1-like 18 DOMAIN: LEA_2	EB438355	0.5	*Arabidopsis thaliana *ATHK1 (histidine kinase 1/ osmosensor)
			EB438355	0.5	*Catharanthus roseus *cold inducible histidine kinase 1 (iK1) mRNA

HC-Pro expression alters significantly (p < 0.05) expression of several genes related to defense and stress responses.

**Fold **change is indicated as a ratio of HC-Pro/WT calculated from normalized median intensity values (n = 3).

**Table 4:** Up- or down-regulation of transcripts in HC-Pro expressing plants

Leaf			Leaf		
EST/mRNA	**Fold**	Description	EST/mRNA	Fold	Description
NP917355	5.1	*Nicotiana tabacum *mRNA for ERF1	TA18922_4097	0.2	*Solanum lycopersicum *CONSTANS 1
FG145666	4.6	*Nicotiana tabacum *RAV mRNA	EB427139	0.2	*Populus nigra *PnLHY2 mRNA for transcription factor LHY
EH620499	3.4	*Arabidopsis thaliana *PLPB (PAS/LOV PROTEIN B)	TA16366_4097	0.3	*Glycine max *MYB transcription factor MYB118 (MYB118) mRNA
AB063574	2.5	*Nicotiana tabacum *WRKY DNA-binding protein	EB434774	0.3	*Arabidopsis thaliana *ATHB-7 (At-HOMEOBOX 7 )
FG637951	2.4	*Nicotiana sylvestris *nserf3 gene for ethylene-responsive element binding 3	EB435512	0.3	*Arabidopsis thaliana *CDF1 (CYCLING DOF FACTOR 1)
DV159714	2.4	*Medicago truncatula *GIGANTEA protein	TA14638_4097	0.4	*Castanea sativa *Late elongated hypocotyl (LHY)
EB433445	2.4	*Arabidopsis thaliana *mRNA for RNA polymerase sigma subunit SigD SIG4 (SIGMA FACTOR 4)	EB428015	0.4	*Nicotiana sylvestris *Ethylene-responsive transcription factor 4 (ERF4)
DW002999	2.2	*Arabidopsis thaliana *KTF1 (KOW DOMAIN CON-TAINING TRANSCRIPTION FACTOR 1)	FG641901	0.4	*Arabidopsis thaliana *basic helix-loop-helix (bHLH) protein
DV162575	2.1	*Arabidopsis thaliana *Transcription initiation factor IIB-2	FG642227	0.4	*Solanum tuberosum *MADS transcriptional factor (Stmads11) mRNA
TA15319_4097	2.0	*Nicotiana tabacum *WIZZ, JA-induced WRKY mRNA	DV999024	0.4	*Populus trichocarpa *SAUR family protein (SAUR23), mRNA, Auxin responsive
**Flower**			TC4480	0.4	*Solanum tuberosum *Jasmonic acid 2, NAC-transcription factor
TA13711_4097	2.6	*Nicotiana tabacum *RAV mRNA	TA17590_4097	0.5	*Oryza sativa *WRKY transcription factor 65 (WRKY65) gene
DV999109	2.3	*Nicotiana tabacum *Ethylene-responsive transcription factor 1 (ERF1)	EB446153	0.5	Tobacco mRNA for TGA1a DNA-binding protein; bZIP transcription factor
TA15319_4097	2.1	*Nicotiana tabacum *WIZZ JA-induced WRKY mRNA	EB424613	0.5	*Camellia sinensis *MYB transcription factor
TA16951_4097	2.1	*Arabidopsis thaliana *ASL37 mRNA for ASYMMETRIC LEAVES2-like 37 protein	DW004709	0.5	*Lycopersicon esculentum *AREB-like protein mRNA; bZIP transcription factor;
AF193771	2.0	*Nicotiana tabacum *DNA-binding protein 4 (WRKY4) mRNA			

HC-Pro expression alters significantly (p < 0.05) expression of several transcription factor genes.

**Fold **change is indicated as a ratio of HC-Pro/WT calculated from normalized median intensity values (n = 3).

### HC-Pro induced differential expression of stress response genes

Pathogen or virus infections in plants induce differential expression of stress responsive genes [15,22]. Our array results indicated differential expression of many genes responsive to cold, salt and dehydration even though the tobacco plants were grown under normal growth conditions (Table 3). In addition, genes in phenyl propanoid pathway (leading from phenylalanine to anthocyanins and lignins) were significantly down regulated (e.g. chalcone synthase and leucoanthosyanidin dioxygenase) [29], whereas terpenoid synthesis (leading from DOXP pathway to carotenoids and brassinosteroids) were significantly up-regulated (e.g. DSX1 and DSX2).

### Altered expression of cell wall biosynthesis related genes in HC-Pro expressing plants

Plant cell wall, the first barrier of defense against invading pathogens, is composed of cellulose microfibrils crosslinked by hemicellulose, pectin, lignin and extensin. Pectins are one of the main components in cell wall against invading pathogens. Endo-polygalacturonase (PG), one of the enzymes secreted at the early stages of infection, depolymerizes the homogalacturonan, the main component of pectin, by cleaving the β-1, 4 glycosidic bonds between the galacturonic acid units [30]. The following oligosaccharides may activate plant defence responses such as synthesis of phytoalexins, lignin and ethylene, expression of proteinase inhibitors and β-1, 3-glucanase and production of reactive oxygen species [31]. Irshad and coworkers [32] have recently provided a new picture of cell wall dynamics in elongating cells by analysing cell wall proteomics and by identifying several new cell wall-related proteins. Interestingly, our microarray reveals that many of these cell wall-associated genes are diffentially expressed in HC-Pro expressing transgenic plants (Table 5). The gene encoding polygalacturonase inhibitor protein precursor (PGIP) was significantly up-regulated [30], whereas the gene encoding polygalacturonase (PG) was down-regulated. Similarily, pectin methyl esterase inhibitor (*PMEI*) transcripts were up-regulated whereas *PME *transcripts were down-regulated in leaves. However, transcripts of *PMEI *were not significantly altered in flowers but transcripts of *PME *and pectin lyases were significantly down-regulated in them (Table 5). Also other genes related to wall dynamics [32], e.g. genes encoding proteases and protease inhibitors (cysteine proteinase, serine carboxypeptidase and trypsin inhibitor), structural proteins (proline rich proteins), and other proteins acting on carbohydrates (alpha-expansins, expansin-like A, chitinase and callose synthetase) were differentially expressed in the HC-Pro expressing plants (Table 5).

**Table 5:** Up- or down-regulation of cell wall related transcripts in HC-Pro transgenic plants

Leaf		
EST/mRNA	Fold	Description
TA16228_4097	6.1	*Nicotiana tabacum *mRNA for DC1.2 homologue, PME inhibitor
FG195661	6.0	*Nicotiana tabacum *cysteine-rich extensin-like protein-4
DV157917	2.7	*Lycopersicon esculentum *xyloglucan endotransglycosylase LeXET2 (LeXET2)
EB425603	2.4	*Solanum lycopersicum *Polygalacturonase inhibitor protein precursor, PGIP
AB176522	2.4	Glucosyltransferase NTGT4 related cluster
AB176524	2.4	*Nicotiana tabacum*, Glycosyltransferase NTGT5b
TA13721_4097	2.4	*Solanum tuberosum *Expansin-like protein precursor
EB444508	2.3	*Phaseolus vulgaris *Hydroxyproline-rich glycoprotein
CV017677	2.1	*Nicotiana tabacum *mRNA for pectin methylesterase
DV160974	0.3	*Nicotiana tabacum *alpha-expansin precursor (Nt-EXPA4) mRNA
EB426691	0.4	*Ricinus communis *cinnamoyl-CoA reductase, putative, mRNA, lignin biosynthesis
TA19759_4097	0.4	*Arabidopsis thaliana *Putative cellulose synthase
FG152217	0.5	*Arabidopsis thaliana *polygalacturonase (PG)
EB424698	0.4	*Arabidopsis thaliana *pectin acetyl estrase
FG179245	0.4	*Arabidopsis thaliana *pectin acetylesterase family protein
**Flower**		
EH623866	3.9	*Solanum lycopersicum *Xyloglucan endotrans-glucosylase-hydrolase XTH3
EB450248	2.3	*Lycopersicon esculentum *Xyloglycan endotransglycosylase precursor
FG644421	2.2	*Ricinus communis *Glycine-rich cell wall protein
EB428200	0.3	*Petunia integrifolia *Pectinesterase precursor
BP133533	0.3	COBRA-like protein 10 precursor related cluster
EB428683	0.3	*Nicotiana tabacum *pectate lyase Nt59
TC6632	0.3	*Arabidopsis thaliana *Cellulose synthase
EB427886	0.3	*Vigna radiata *Pectinacetylesterase precursor
FG155250	0.3	*Nicotiana tabacum *pectin methylesterase (PPME1) mRNA

Transcripts encoding pectin, lignin and extensin synthesis and degradation were altered significantly (p < 0.05) in HC-Pro transgenic plants.

**Fold **change is indicated as a ratio of HC-Pro/WT calculated from normalized median intensity values (n = 3).

### Flowering time is delayed in HC-Pro transgenic plants

Transgenic HC-Pro expressing plants had a late flowering phenotype when compared to wild type tobacco plants. Therefore, it was not surprising that expression of the circadian clock genes and genes involved in flower induction was altered in the HC-Pro expressing transgenic plants [33,34]. The genes encoding a blue light receptor FKF1, GIGANTEA and PLPB, a PAS/LOV protein, were all up-regulated, whereas the gene encoding CYCLING DOF FACTOR1 (CDF1) protein for the induction of CONSTANS (CO) gene was down-regulated (Table 6). The down-regulation of the *CO *gene may have affected the regulation of photoperiodic *FLOWERING LOCUS *(*FT*) gene and caused the late flowering phenotype. Moreover, EARLY flowering 4 (ELF4) is also known to regulate oscillatory properties of circadian clock, and it's over expression induces late flowering phenotype under long day conditions in *Arabidopsis *[35]. The *ELF4 *transcripts were also clearly up-regulated in HC-Pro expressing plants (Table 6). Also several AP2-related transcription factors were up-regulated both in leaf and flower tissues, i.e. ERF1 and RAV2 (also known as TEMPRANILLO [36].)

**Table 6:** The expression of circadian and flowering time related genes in transgenic HC-Pro plants

Leaf		
EST/mRNA	Fold	Description
FG637943	14.2	*Arabidopsis thaliana *FKF1 (FLAVIN-BINDING, KELCH REPEAT, F BOX 1); signal transducer/ two-component sensor/ ubiquitin-protein ligase (FKF1) mRNA
DV161898	5.1	*Arabidopsis thaliana *zinc finger (B-box type) family protein (AT1G68520) mRNA
FG145666	3.4	*Nicotiana tabacum *RAV mRNA
EH620499	3.1	*Arabidopsis thaliana *PLPB (PAS/LOV PROTEIN B)
BP531560	2.6	*Solanum lycopersicum *Putative EARLY flowering 4 (ELF4) protein
DV159714	2.4	*Medicago truncatula *GIGANTEA protein
TA18922_4097	0.2	*Solanum lycopersicum *CONSTANS 1
EB427139	0.2	*Populus nigra *PnLHY2 mRNA for transcription factor, LHY; SANT 'SWI3, ADA2, N-CoR and TFIIIB- domains
EB680212	0.3	*Solanum tuberosum *cultivar Early Rose CONSTANS mRNA
EB435512	0.3	*Arabidopsis thaliana *CDF1 (CYCLING DOF FACTOR 1)
TA14638_4097	0.4	*Castanea sativa *Late elongated hypocotyl (LHY)

Statistical significance of up- or down-regulated genes was tested using Student's t-test (p < 0.05).

**Fold **change is indicated as a ratio of HC-Pro/WT calculated from normalized median intensity values (n = 3).

### Proteases and proteosomal degradation

HC-Pro protein of PVY has a special function in cleaving the polyprotein into functional viral proteins. Based on its functional domains the HC-Pro protein is characterized as a cysteine-type endopeptidase and thioredoxin. However, it is not known whether expression of this protein in transgenic tobacco plants could induce proteolytic activity and reversible oxidation of two cysteine thiol groups in tobacco cells. Our microarray data showed that several genes encoding protease inhibitors were induced suggesting that this response was induced to resist protease and proteasomal degradation (Table 7). These included trypsin and metallocarboxy-peptidase IIa proteinase inhibitors, and many of these proteases are known to be cell wall-associated proteins [32,37]. Also proteasome-related genes like ubiquitin ligases were induced.

**Table 7:** Up- or down-regulation of genes involved in protein degradation by proteases or proteosomal machenery in transgenic HC-Pro plants

Leaf		
EST/mRNA	Fold	Description
EB438380	27.5	*Solanum lycopersicum *unknown trypsin inhibitor-like protein precursor
FG637943	14.2	*Arabidopsis thaliana *FKF1 (FLAVIN-BINDING, KELCH REPEAT, F BOX 1)
TA13877_4097	10.9	*Nicotiana glutinosa *putative proteinase inhibitor mRNA
TA12601_4097	4.6	*Acyrthosiphon pisum *ubiquitin ligase E3
CV019298	3.3	*Solanum tuberosum *metallocarboxypeptidase inhibitor IIa
CV018626	3.0	*Ricinus communis *Serine carboxypeptidase, putative, mRNA, AT3 g45010/F14D17_80
FG643489	2.8	*Arabidopsis thaliana *AtPP2-B13 (Phloem protein 2-B13); carbohydrate binding F-box protein 3
TA17751_4097	2.6	Development and cell death domain, the KELCH repeats and ParB domain.
CV018465	2.4	Subtilisin-like protease related cluster
BP133434	2.4	*Ricinus communis *protein binding protein, putative, mRNA (ubiquitin protein ligase)
TA17959_4097	2.3	*Mirabilis jalapa*, ubiquitin ligase
FG635491	2.1	*Ricinus communis *RING-H2 finger protein ATL2B, putative, mRNA
BP530000	2.1	Kelch repeat-containing F-box protein-like
FG137301	2.1	Tomato ATP-dependent protease (CD4A)
TA18536_4097	0.2	*Arabidopsis thaliana *LKua-ubiquitin conjugating enzyme, F19K23.12 protein
CV019784	0.4	*Lycopersicum esculentum *mRNA for serine protease, SBT1
EB429242	0.4	LIM, zinc-binding; Ubiquitin interacting motif; Peptidase M, neutral zinc metallopeptidases,

Statistical significance was tested using Student's t-test (p < 0.05).

**Fold **change is indicated as a ratio of HC-Pro/WT calculated from normalized median intensity values (n = 3).

### Gene expression related to photosynthesis

**Figure 2:**
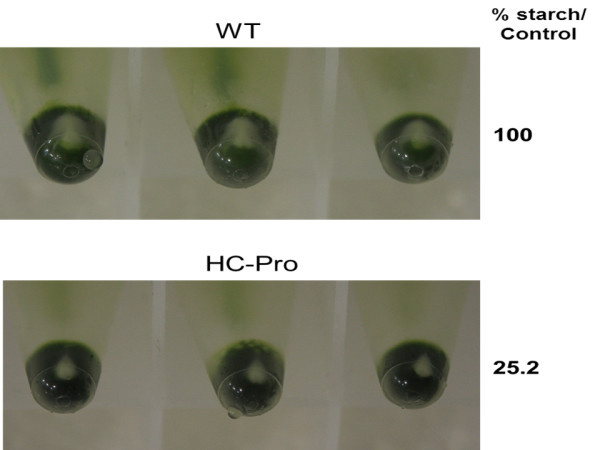
**Starch granules at the bottom of Eppenforf tube pelleted during thylakoid preparation**. For each of thylakoid isolation, 1 g of wild type (WT) or transgenic HC-Pro (HC-Pro) leaves (fresh weight, FW) was used. Three biological replicates are presented in the figure. The amount of starch was also quantified after removing the soluble sugars (on the right). The quantification of starch indicated about four-times less starch in HC-Pro expressing leaf samples than in wild type tobacco leaf samples (n = 4).

Microarray results also indicated altered gene expression for enhancing energy production (ATP). Mitochondrial and chloroplastic ATP synthetase genes were both up-regulated. Genes encoding starch degradation (alpha-amylase and alpha-glucan water dikinase) were up-regulated, and genes encoding starch synthesis were down-regulated (Table 8). These results are related to the reduced amount of starch granules in leaves of the HC-Pro expressing plants, and this was confirmed by visual observation of starch pellets during thylakoid preparation (Figure 2), and by direct quantitation of starch from the leaf samples (Additional file 7). Genes involved in glycolysis, like phosphoenolpyruvate (*PEP*) carboxylase and its activating kinase were also down-regulated. Sugar transporters and isomerases were up-regulated, whereas sucrose-phosphate synthase (*SPS*) gene was down-regulated. In addition, carbon assimilation-related genes large subunit of rubisco (*RBCL*) and rubisco subunit binding beta were both up-regulated. At the same time, the genes encoding chlororespiration (CCR4) and cyclic electron transport proteins (PGR5) were clearly down-regulated possibly allocating more electrons to linear electron transport. All these sugar metabolism-related gene expression alterations imply carbon metabolism imbalance, and indicate higher glucose over sucrose content in cells.

**Table 8:** Expression of photosynthesis, sugar metabolism and S-adenosyl methionine biosynthesis related transcripts in transgenic HC-Pro plants

Leaf			Leaf		
EST/mRNA	Fold	Description	EST/mRNA	Fold	Description
EH620909	3.6	*Nicotiana tabacum *photosystem I reaction center subunit (PsaN) mRNA	NP916903	3.6	*Nicotiana tabacum *asparagine synthetase (DARK INDUCIBLE 6) (DIN6) mRNA
EB427609	3.5	Rubisco subunit binding-protein beta subunit-like	EH618866	2.2	*Arabidopsis thaliana *ADENINE PHOSPHORIBOSYL TRANSFERASE 1 (APT1) mRNA
FG146265	2.8	*Solanum tuberosum *alpha-glucan water dikinase (SEX1)	FG176614	2.0	*Arabidopsis thaliana *DIN10 (DARK INDUCIBLE 10)
TA22161_4097	2.8	*Nicotiana sylvestris *ATP synthase subunit beta, chloroplastic	FG140432	2.6	*Nicotiana benthamiana *asparagine synthetase (DIN6) mRNA
TA12737_4097	2.7	*Nicotiana plumbaginifolia *ATP synthase subunit alpha, mitochondrial	EB435670	0.3	*Arabidopsis thaliana *NRT1.5 (NITRATE TRANSPORTER) mRNA
TA11967_4097	2.6	*Nicotiana sylvestris *Ribulose bis-phosphate carboxylase large subunit	TA12496_4097	0.4	*Solanum tuberosum *Granule-bound starch synthase 1, chloroplast precursor
DQ460148	2.6	*Solanum tuberosum *glucose-6-phosphate/phosphate translocator 2	CV017874	0.4	*Nicotiana langsdorffii × Nicotiana sanderae *sucrose-phosphate synthase 2 (SPS) mRNA
EB681343	2.4	*Nicotiana tabacum *ATP synthase alpha chain	TA13160_4097	0.4	*Solanum tuberosum *Adenylate kinase family-like protein
BP128932	2.4	*Arabidopsis thaliana *CRR4 (CHLORORESPIRATORY REDUCTION 4)	AY741503	0.4	*Nicotiana tabacum *S-Adenosyl- L-methionine methyltransferase (SAMT) mRNA
EB102906	2.4	*Actinidia chinensis *Plastid alpha-amylase	EB429936	0.5	*Lycopersicum esculentum *S-adenosyl-L-methionine synthetase mRNA
DV159621	2.4	*Nicotiana tabacum *NADPH: protochlorophyllide oxidoreductase			
EB426704	2.3	*Arabidopsis thaliana *sugar isomerase (SIS8)			
EH622880	2.2	*Nicotiana tabacum *CP12 precursor			
CV021666	2.2	*Nicotiana tabacum *chloroplast post-illumination chlorophyll fluorescence increase protein mRNA			
AJ001771	2.1	*Nicotiana tabacum *Glucose-6-phosphate dehydrogenase			
DV160944	2.0	*Spinacia oleracea *Ribose-phosphate pyrophosphokinase 4			

Statistical significance was tested using Student's t-test (p < 0.05).

**Fold **change is indicated as a ratio of HC-Pro/WT calculated from normalized median intensity values (n = 3).

**Figure 3:**
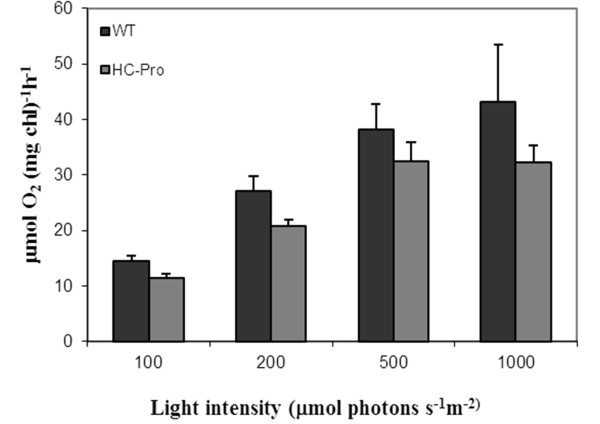
**Light-responsive O_2_-evolution of photosystem II was measured of wild type (WT) and HC-Pro expressing plants**. O_2_-evolution was measured of freshly isolated thylakoid membranes using DCBQ as an electron acceptor. Standard error of mean is presented as bars abobe the columns (n = 6, consisting of three biological and two technical replicates).

As there were clear changes in expression of genes encoding proteins involved in the photosynthetic light and dark reactions, some photosynthetic parameters were measured. Light responsive curve of photosystem II (PSII) activity (Figure 3) indicated decreased PSII oxygen evolution activity in HC-Pro expressing leaves. On the other hand, the reduced amount of starch in leaves of the HC-Pro expressing plants may also indicate problems in carbon fixation in chloroplast stroma (Figure 2).

It is well documented that carbon metabolism affects gene expression [38,39]. Our results indicated that many dark induced (*DIN*) genes as well glucose/sucrose regulated genes were differentially regulated in HC-Pro expressing plants. E.g. asparagine synthetase (*DIN6*) and α-amylase genes were up-regulated and *SPS*, nitrate reductase (*NR*) and adenylate kinase (*AMK*) genes were down-regulated (Table 8). Also the genes encoding sugar balance sensor molecules were differentially regulated. The histidine-kinase 1 like (*ATHK1*-like) gene involved in water balance sensing and dehydration was down-regulated, whereas the *SNF1-RELATED PROTEIN KINASE *(*SNRK*) gene involved in sugar metabolite stress-responsive gene regulation was up-regulated in the HC-Pro expressing plants (Table 3).

As metabolism-related gene expression suggests energy (ATP) depletion in HC-Pro expressing plants, a high AMP/ATP ratio is expected. This probably affects several ATP demanding processes like production of SAM [40,41]. Recycling of adenosine is of vital importance in this process. However, we did not detect any changes in the expression of gene encoding adenosine kinase (ADK), but instead we detected change in expression of two genes encoding enzymes equilibrating adenine nucleotides, namely *AMK *and Ade phosphoribosyltransferase (*APT*) (Table 8). *AMK *transcripts were down-regulated whereas *APT *transcripts were up-regulated in HC-Pro expressing leaves. These both affect balance between adenine, AMP and ADP. In addition, genes encoding SAM synthase and transferase (SAMT) were clearly down-regulated (Table 8). SAM is the key compound for all transmethylation reactions like methylation of pectin, DNA, RNA, histones and polyamine synthesis. Moffatt et al. 2002 [40] have created adk sense and antisense mutant lines to inactivate ADK enzyme in transgenic *Arabidopsis *and found both developmental abnormalities (a compact, bushy appearance of plants with small, rounded and waxy leaves) and reduced transmethylation activities (e.g reduced level of methylation of polygalaturonic acid) in these plants. The phenotype of these transgenic lines correlates well with our HC-Pro expressing tobacco plants indicating the central role of the transmethylation reactions in the plant development and differentiation.

### Protein profiles are strongly altered

**Figure 4:**
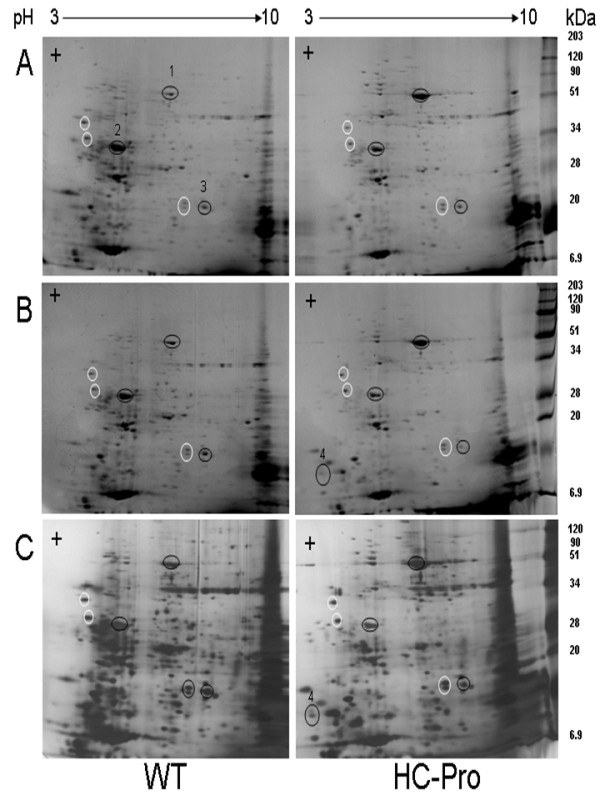
**Proteome analysis of two biological replicates of wild type (WT) and HC-Pro expressing plants (HC-Pro)**. Proteins isolated from leaves were separated by using 2D-polyacrylamide gel electroforesis (2D-PAGE). Proteins in two isoelectric focused strips (WT and HC-Pro) were separated the second dimension in a large SDS-polyacrylamide gel. Upper gels (A and B) are stained using colloidal coomassie blue and the lower gel (C) using silver staining. White circles indicate control protein spots, whose intensity was not changed and black circles indicate protein spots that were either increased (1, RBCL and 4, PsaN, CP12) or decreased (2, OEE33 and 3, CYP2) in HC-Pro expressing plants. The identity of numbered protein spots was analysed using LC-ESI MS/MS mass spectrometry.

Both the energy deficiency and the altered transcript levels affect the level of protein synthesis. Therefore quantitative changes in the proteome were analysed using 2D-PAGE from the same leaf samples that were previously analysed in microarray. Results indicated dramatic changes in the protein composition between the wild type and HC-Pro expressing plants (Figure 4). A few spots of distinctly up- or down-regulated proteins, as visualized in the 2D-acrylamide gels, were analysed by peptide sequencing after trypsin cleavage using LC-ESI MS/MS mass spectrometry. All identified protein spots turned out to be related to photosynthesis. The first identified, strongly up-regulated spot was RBCL. The transcript of the gene *RBCL *was also up-regulated in leaves of the HC-Pro expressing plants (Table 8). Second identified spot was oxygen-evolving enhancer protein 1 (OEE33, gene name *psbO*). Even though this protein was clearly down regulated, *psbO *was not found in the list of up- or down-regulated transcripts, while other transcripts encoding thylakoid lumen proteins (*psbP *encoding a 29.8 kDa protein and *psbS *gene encoding a 22 kDa protein) were found to be down-regulated. Third analysed, down-regulated spot was identified as tobacco CYP2 protein. This 20 kDa protein has a high homology with AtCYP20-2 protein [42]. These peptidyl-prolyl cis-trans isomerases (PPIase) are redox-dependent proteins catalyzing folding of proteins in the thylakoid lumen of plant chloroplasts. Two chloroplast-directed tobacco proteins were identified in the fourth analysed, up-regulated spot; a 12 kDa chloroplast protein (CP12) and a photosystem I reaction center subunit (PsaN). The gene encoding for PsaN protein was also the most up-regulated gene in the list of photosynthesis-related genes (Table 8).

## Discussion

This study provides a comprehensive picture of transcriptional changes in tobacco leaves and flowers due to expression of HC-Pro RSS derived from PVY. As far as we know this is the first systemic analysis of viral RSS-induced gene expression alterations in tobacco host. HC-Pro RSS interferes with the silencing machinery.

The full genomic sequence of tobacco is not known, which limits the systemic analysis of transcriptional profiles in this species. However, a large collection of various EST and mRNA data is available and has been applied to construct a 44 000 element microarray (Agilent) that provides the best possible approach for the systemic study of the tobacco gene functions today.

Previously, accumulation of small RNA pools have been systemically analysed via deep sequencing projects [43-45]. Expression of viral RSS in transgenic plants have been shown either to decrease the amount of miRNAs, or to reduce the activity of the silencing processes, which should lead to increase of the specific miRNA-regulated target mRNAs. However, these regulatory defects seem to lead often to complex cascades of effects. MacLean & coworkers [46] have shown that silencing-mediated regulatory reactions are highly interconnected and back-regulated and form intensive and multilayered regulatory networks. Indeed, we found in the list of genes modulated in our experiments many mRNAs that has been previously shown to contain target sites for miRNAs [43] and thus be post-transcriptionally regulated. The microarray analysis indicated that the expression levels of multiple genes (748 genes in leaves and 332 genes in flowers) were significantly altered in HC-Pro expressing transgenic plants.

### Defence and stress response in HC-Pro expressing plants

The expression of HC-Pro RSS induced similar changes in gene expression profile as has been detected in virus infected plants [15,26]. We found that genes related to defence and both biotic and abiotic stress responses (jasmonic acid and ethylene responsive genes), transcriptional regulators (e.g. ERFs, RAV2), protein degradation related (proteasomal) proteins and proteases, and genes involved in photosynthetic reactions were altered in HC-Pro expressing tobacco plants in similar way as in *Arabidopsis *plants infected either by a TEV or CMV-Y [15,22,23,25]. The reason for this might be that the virus encoded RSSs interfere with long silencing mediated regulatory cascades, and their affects can be amplified through extensive regulatory networks. In conclusion, the expression of *HC-Pro *gene alone largely simulates the effects of a virus infection in plants, indicating that it is a major factor in viral pathogenicity.

HC-Pro RSS induced a general defense and stress response (e.g. PR-proteins) in transgenic tobacco plants (Tables 3). Liang et al. [47] have also shown that B3-subgroup of AP2 transcription factors (ERF1, ERF3) regulates expression of pathogenesis-related genes (PR). We found these transcription factors up-regulated in both tobacco leaf and flower samples, which apparently lead to activation of other stress response genes. Salt, low temperature and dehydration responsive genes were induced, even thought the plants were not suffering from any kind of stress conditions. However, these stress responses might be also due to secondary effects from other primary causes, e.g. defects in photosynthetic light reactions and carbon metabolism, leading to shortage of sugar molecules comparable to cold or dehydration stress conditions [48].

### Phenotypic changes related to changed gene expression

The phenotypic changes found in transgenic HC-Pro expressing plants were induced most probably by changed expression of genes that regulate developmental differentiation. HC-Pro suppresses the activity of miRNAs, thus chancing the normal post-transcriptional regulation of various transcription factors that regulate developmental timing (flowering) and other developmental processes (leaf structure, stem internodes). Recently, Imaizumi [49] has reviewed genes involved in circadian clock and photoperiodism in *A. thaliana*, and their regulation by RNA-silencing. It has been previously shown that AP2 transcription factors (TOE1-3) are regulated by miR172, and a late-flowering mutant was produced by constitutive expression of the miR172 target gene *TOE1 *[50]. Many AP2-related transcription factors (e.g. ERF1 and RAV2) were enhanced in the HC-Pro expressing plants, possibly due to HC-Pro-mediated suppression of the miR172 function. It seems that miR172 is also regulated further by miRNA, namely miR156 [51].

Our results indicated that up-regulation of two ethylene responsive transcription factors (*ERF1 *and *RAV2 (TEMPRANILLO)*) may have caused differential expression of defense-related genes and late flowering phenotype, respectively [27,36,47,49,51,52]. Late flowering phenotype in plants may be also due to problems in measuring the day length, which may induce problems in shifting from vegetative to reproductive phase of growth [33-35]. Expression of the whole set of genes encoding blue light receptors, transcription factors and proteasomal E3-ligases, all involved in induction of the flowering time locus (*FT*) were altered (Table 7). The altered regulation of these genes in HC-Pro expressing plants may have postponed the plant's normal flowering time induction.

### The cysteine endopeptidase and thioredoxin properties of the expressed HC-Pro may also affect the protein profile

Various characterized viral RSSs have different functional mechanisms [3,5,53-55]. In addition most of these proteins mediate also other functions which are essential for the viral life cycle and pathogenicity. Potyviral HC-Pro protein has domains of a cysteine endopeptidase and thioredoxin that may function in degradation of proteins containing cysteine residues, and changing the redox state of proteins (reduction of disulfide bonds to reduced cysteines) and these activities may have also contributed to the primary responses of HC-Pro expressing plants. Photosynthesis is the source of all the energy in plants by sugar metabolism, and it is known to be tightly regulated by redox states of the chloroplast proteins. The thioreduction of these proteins in cytoplasm could easily impair photosynthesis, and thereby lead to sugar starvation and further on, to altered regulation of the metabolic stress-related genes. The general stress response observed in the HC-Pro expressing plants can thus be due to direct alterations in the expression levels of some vital genes, and/or due to secondary effects, which again can be mediated by silencing suppression, cysteine endonuclease, thioreduction, impaired proteasomal functions or by all of those mechanisms. It appears that the disturbance of the normal chloroplast functions plays a central role in these response cascades.

The microarray data suggests that HC-Pro expressing tobacco cells have energy shortage, and the up-regulation of *DIN *genes might be one symptom of this. Normally, the *DIN *genes are induced under dark treatment or by various sugar metabolism defects [38,39], and both photosynhetic light and dark reactions are involved in regulation. Light activation curve of O_2_-evolution indicated decreased photosynthetic capacity in HC-Pro expressing transgenic plants (Figure 3), and the proteomic data also indicated that these plants indeed have problems in oxygen evolution in their PSII reaction center. To compensate this defect, genes encoding carbon fixation enzymes (RBCL and Rubisco subunit binding protein) were up-regulated. Also the production of storage sugar molecules was affected, as starch degradation was enhanced and synthesis reduced in HC-Pro expressing leaves (Table 8 and Figure 2). In addition, glycolysis was not used to gain energy from sugar molecules (e.g. repression of genes encoding PEP carboxylase and its activating kinase).

The genes regulating ATP synthesis in mitochondria and chloroplasts were clearly up-regulated, which may explain why many ATP-demanding systems, such as translation, were strongly altered (Figure 4). Another energy-dependent key process is the production of SAM [40], which is a general donor of methyl groups in the transmethylation reactions both in cytosol and in chloroplasts and mitochondria. A gene encoding plastid membrane-located SAMT protein was down-regulated more than two times in HC-Pro expressing plants, thus possibly affecting SAM levels in the chloroplasts, chloroplast biogenesis, and methylation reactions in chloroplasts [56]. High level of SAM is also needed for pectin synthesis of cell walls. Pectin is transported as highly methylated molecule into cell wall and must be demethylated by PME prior to insertion to cell wall. Due to decreased transmethylation capacity, the cell wall and especially pectin synthesis may have been affected.

The up-regulated *PMEI *and *PGIP *transcripts are both shown to be involved in resistanse against pathogenic attacks. An and co-workers [57] have recently shown that the PMEI is required for antipathogenic activity, basal disease resistance and abiotic stress tolerance, and that PMEI is clearly up-regulated in these biotic and abiotic stresses, and also by treatments with ethylene and jasmonic acid. Interestingly, PME is also known to be involved in viral *tobacco mosaic virus *(TMV) movement by binding to movement protein (MP) and assisting movement of viruses from cell to cell [58-60].

## Conclusions

Multiple gene functions are affected in the HC-Pro expressing transgenic plants, and these alterations induce a high stress status to the cells. Many of these stress responses appear to be interconnected, so that some to them are direct, but some are indirect, either caused by altered regulation of important transcription factors, induced by products of various signaling pathways i.e. ethylene and jasmonic acid pathways, or via the altered redox state of the cells. It appears that the sole HC-Pro protease/silencing suppressor protein can off-set the cellular regulatory network very drastically. Surprisingly the transgenic plants can still differentiate to fairly normal (even if malformed), seed producing phenotypes, indicating that the buffering capacity and redundancy of the genetic regulation is amazingly strong.

## Methods

### Plant material

The wild type tobacco (*Nicotiana tabacum*) and transgenic tobacco plants expressing *HC-Pro *transgene [10] were grown in greenhouse conditions at 60% relative humidity and 22°C, with a day/night regime of 16 h light (150 μmol photons m^-2^s^-1^) and 8 h dark. Leaf samples (third leaf from the top) were taken from one-month-old plants, the plants were at that time about 20 centimeters of height. Leaf and flower samples were taken from the same plant. Flower samples were taken one day prior to opening. Both leaf and flower samples were directly frozen in liquid nitrogen and stored at -80°C.

### RNA extraction, cDNA labeling and microarray hybridization

Total RNA was isolated from leaves and flowers of wild type and transgenic plants using TRIsure-reagent (Bioline, UK) according to manufacturer's recommendations. Total RNA was further purified using RNeasy clean up column (QIAGEN inc. USA).

The cDNA labeling was performed using Agilent's Quick Amp Labeling kit for one-color (Product number 5190-0442). 700 ng of purified total RNA was used to produce the Cy3 labelled cDNAs. All samples were processed together with Agilent's RNA spike kit (Product number 5188-5282). The quality of total RNA and labelled cDNA was checked using Agilent's 2100 bioanalyzer RNA 6000 Nano kit (Product number 5067-1511). The concentration of Cy3 labelled cDNA was also measured using NanoDrop ND-1000 spectrofotometer. 1.65 μg Cy3 labelled cDNA was hybridized on a Agilent's 4 × 44 K tobacco chip (Design ID 21113) at 65°C over night (17,5 h) using solution provided in Agilent's Gene Expression Hybridization kit (Product number 5188-5242) according to manufacturer's recommendations.

The chips were washed after hybridization using ready-made solutions in Agilent's Gene Expression Wash Pack (Product number 5188-5327), in which the 0.005% Triton X-102 was added according to manufacturer's recommendations. The chips were further treated with Agilent's Stabilization and Drying solutions (Product number 5190-0423). The chips were scanned using Agilent Technologies Scanner, model G2565CA. Numeric data was produced using Agilent Feature Extraction software version 10.5.1.1. Grid: 021113_D_F_20080801; Protocol: GE1_105_Dec08; QC Metric Set: GE1_QCMT_Dec08.

The raw numerical data obtained after scanning microarray chips was analysed by using the R Project for Statistical Computing program ([61], Agi4 × 44 k preprocess, Lopez-Romero, 2010). In order to compare intensity values of different samples (control, (6) vs. transgenic plant samples, (3)), the leaf samples were normalized together and the flower samples were as well normalized together. Normalization of three biological replicates was performed using median signal values and median background values. A background offset value (50) was added to prevent negative values during normalization. Normalization of the arrays was performed using a "quantile" parameter. All data handling was performed using Chipster, a visual program based on R Project for Statistical Computing program (Center of Scientific Calculating (CSC), Finland). The array results have been deposited into ArrayExpress with accession number E-MEXP-3105.

### Re-annotation of differentially regulated gene elements of 44 k tobacco chip

The tobacco genome is not totally sequenced like *A.thaliana*; instead the 44 k tobacco chip is based on known tobacco genes, but also not so well annotated EST and cDNA sequence information. The differentially regulated genes that were up- or down-regulated more then two times in our tobacco 44 k array were re-annotated using three different methods to get a proper functional annotation for the unknown gene names. In the first method the cDNA sequence was looked for similar DNA sequence using NCBI Blast Search. In the second method the cDNA sequence was translated to protein sequence (ExPASy-translate tool, SIB; Swiss Institute of Bioinformatics) and then homologous proteins were searched using FASTA/SSEARCH/GGSEARCH/RCH - Protein Similarity Search (EMBL-EBI). In the third method, larger cDNAs were searched from Plant Transcript Assemblies Database (TIGR). Different tobacco EST and cDNA sequences are assembled to larger over-lapping cDNA sequences increasing the quality of annotation against other known plant cDNAs. Using these three methods, reliable annotation for most differentially regulated genes was obtained.

### Verification of differentially expressed genes

The array results were verified by using RT-qPCR according to MIQE guidelines [62]. The RT-qPCR was performed from the same RNA samples as were previously used in microarray experiments. The cDNA was synthesized from 1 μg of purified leaf or flower total RNA using RevertAid H-Minus M-MuLV reverse transcriptase according to manufacturer's recommendations (Product # EPO451, Fermentas). Produced cDNA was diluted 1:15 and 3 μl was used in RT-qPCR (Maxima SYBR Green/Fluorescein qPCR MasterMix (2X) (Product # KO242, Fermentas). The gene specific reference and sample primers used in RT-qPCR are listed in Additional file 1. For each three biological replicates, three-four technical replicates were run to minimize pipetting errors. RT-qPCR reactions were run in a 96-well plate containing both wild type (reference) and HC-Pro transgenic samples. The RT-qPCR was performed using Bio-RAD's iQ5 machine. The results were calculated using the quantification cycle (C_q_) method (delta delta Cq) according to Bio-RAD's iQ5 default settings (see [62]). All primer pairs produced only one peak in DNA melting curves indicating high specificity of the primers. Standard error of mean (s.e) was also calculated of three biological replicates.

### Photosynthetic measurements

Equal amount of intact wild type and *HC-Pro *transgenic tobacco leaves (1.0 g) were ground in an ice cold mortel in 4 ml of thylakoid isolation buffer (0.3 M sorbitol, 50 mM Hepes/KOH pH 7.4, 5 mM MgCl_2_, 1 mM EDTA and 1% BSA). Suspension was filtered through a Miracloth and 2 ml thylakoid suspension was pelleted in Eppendorf-centrifuge 12 000 × g for 2 minutes (a picture was taken of thylakoids with a starch pellet, see Figure 2). The amount of starch was also quantified by using Megazyme total starch assay procedure (see details in Additional file 7). The pellet was resuspended into 100 μl of O_2_-electrode measuring buffer (0.3 M sorbitol, 50 mM Hepes/KOH pH 7.4, 5 mM MgCl_2_, 1 mM KH_2_PO_4_). Oxygen evolution was measured directly in a Clark type O_2_-electrode using 0.5 mM DCBQ as electron donor. The chlorophyll concentration was calculated according to Porra et al. [63]. Samples in the cuvette were quantified based on equal amount of total chlorophyll.

### Isolation of proteins, 2D-PAGE and Western blotting

Protein samples of leaves from wild type and HC-Pro expressing transgenic plants were isolated concurrently with the RNA isolation using TRIsure-reagent (Bioline). The protocol was adapted from TRIzol (Invitrogen inc. USA) and performed according to manufacturer's recommendations. The protein concentration was measured using Lowry method. Proteins were first separated by Bio-Rad laboratories 7 cm IPG strips pH 3-10 according to manufacturer's recommendations. 250 μg of protein was loaded per a strip. Strips containing wild type and transgenic HC-Pro focused protein samples were then run simultaneously in a large gel in Protean II apparatus (Bio-Rad) to produce a similar mobility of focused proteins of both strips. Protein gels were then fixed and stained in colloidal Coomassie blue stain (PageBlue staining kit, Fermentas) according to manufacturer's recommendations, destained and photographed. Some of the gels were also stained a second time with silver stain (PageSilver silver staining kit, Fermentas). Selected protein spots were taken from PageBlue stained gels and the protein identity was analysed after trypsin treatment using LC-ESI-MS/MS mass spectrometry in the proteomics unit (Turku Centre of Biotechology). Samples were analysed using Qstar i (Applied Biosystems/ MDS Sciex), coupled with a CapLC HPLC machine (Waters). Peptides were first loaded into pre-column (0.3 × 5 mm PepMap C18, LC Packings) and then peptides were separated in a 15 cm C18 column (75 μm × 15 cm, Magic 5 μm 100Å C18, Michrom BioResources Inc. Sacramento, CA, USA) using a 20 min gradient. Peptide sequence search was performed using a Mascot program (v2.2.6) UniProt (release 2010_9) (see Additional files 8, 9, 10 and 11).

## Authors' contributions

AJS grew and collected the plant material for the experiments. The experimental work and the reannotation of significantly altered trancripts were carried out by AJS and BJ together. AJS wrote and KL proof read the manuscript. All authors have read and approved the manuscript.

## Supplementary Material

Additional file 1 **Primers for RT-qPCR and a table representing expression of HC-Pro mRNA in transgenic plants measured by using RT-qPCR**. Click here for file

Additional file 2 **Supplemental Table 2**. Normalized leaf microarray data Click here for file

Additional file 3 **Supplemental Table 3**. Normalized leaf microarray data with FDR Click here for file

Additional file 4 **Supplemental Table 4**. Normalized flower microarray data Click here for file

Additional file 5 **Supplemental Table 5**. Normalized flower microarray data with FDR Click here for file

Additional file 6 **Supplemental Figure 6**. A BOX-PLOT presentation of data based on Supplemental Tables 3 and 5 Click here for file

Additional file 7 **Method and results of starch quantification**. Click here for file

Additional file 8 **Supplemental Table 8**. Peptide sequencing spot 1 (see Figure 4) Click here for file

Additional file 9 **Supplemental Table 9**. Peptide sequencing spot 2 (see Figure 4) Click here for file

Additional file 10 **Supplemental Table 10**. Peptide sequencing spot 3 (see Figure 4) Click here for file

Additional file 11 **Supplemental Table 11**. Peptide sequencing spot 4 (see Figure 4) Click here for file
