# Supplementary material for: HC-Pro silencing suppressor significantly alters the gene expression profile in tobacco leaves and flowers
Source: BMC Plant Biol. 2011 Apr 20;11:68. doi: 10.1186/1471-2229-11-68 (PMC3111369; doi:10.1186/1471-2229-11-68)
Supplement: Additional file 1 — Primers for RT-qPCR and a table representing expression of HC-Pro mRNA in transgenic plants measured by using RT-qPCR. [file 1471-2229-11-68-S1.DOC]

**Additional file 1** Primers used in RT-qPCR and a table representing expression of HC-Pro mRNA in transgenic plants measured using RT-qPCR.

Primers for reference genes:

EB450395; Nicotiana tabacum ARPC3 (actin-related protein C3)

LEFT PRIMER CTGCAGAGGGTGATTTTGCT

RIGHT PRIMER ATTGTGCATCACCAGCCATT

SEQUENCE SIZE: 84

X67159; Nicotiana tabacum Nicotiana tabacum pectate lyase mRNA

LEFT PRIMER TGGGAAAGGTACGCTATTGG

RIGHT PRIMER TCGCACCATTTTCCATATCA

SEQUENCE SIZE: 184

Primers for differentially expressed genes:

TG-HcPro; Primer pair for testing HC-Pro expression

LEFT PRIMER TGGGGGTTATGGATTCAATG

RIGHT PRIMER CATTATCGCTGCAACTCTGC

SEQUENCE SIZE: 152

AY772945; Nicotiana tabacum pectin methylesterase mRNA, complete cds

LEFT PRIMER TTTCGTGTTTGGTGATGCAG

RIGHT PRIMER GAAACGCCTTCATTTGTGGT

SEQUENCE SIZE: 197

FG156808; Nicotiana tabacum P-rich protein NtEIG-C29

LEFT PRIMER CATCTGGCTTCCAGTGTCCT

RIGHT PRIMER CGTCCCCTAGAAAACCCTGT

SEQUENCE SIZE: 287

FG157361; Nicotiana tabacum RAV mRNA

LEFT PRIMER GCCCTTGCTTGAAAATGAAG

RIGHT PRIMER GGGAAATGTTTTTCAGCATGT

SEQUENCE SIZE: 404

EB438380; Solanum lycopersicum Trypsin and protease inhibitor

LEFT PRIMER CCTAGTGCCACATCACGTTG

RIGHT PRIMER GCAATACATTGCTGGGAGTTT

SEQUENCE SIZE: 428

EH620344; Arabidopsis thaliana FKF1

LEFT PRIMER TCTTGCTGTTTGTAGTCCCTTG

RIGHT PRIMER AGAATATATAAATACAACGGCAAAAAG

SEQUENCE SIZE: 166

EH615198; Nicotiana tabacum nictaba (NT1)

LEFT PRIMER GGCTTCGATATTCGTCCAAA

RIGHT PRIMER TCACATGCACTTACAACAAGCTC

SEQUENCE SIZE: 211

FG156808; Nicotiana tabacum 1-D-deoxyxylulose 5-phosphate synthase

LEFT PRIMER CCTAGAGGAAATGGAGTTGGTG

RIGHT PRIMER GATGTCTGCAGCTCCCAAA

SEQUENCE SIZE: 210

Nicotiana tabacum S-Adenosyl-L-methionine methyltransferase (SAMT)

AY741503

LEFT PRIMER AAGCATGGGTTTCAATCACC

RIGHT PRIMER ACAACCAACTCATTGAGGGC

SEQUENCE SIZE: 228

| Expression of HC-Pro mRNA in transgenic plants measured using RT-qPCR. Numbers are indicating quantification cycle (C_q_) values. No PCR products were detected in wild type samples (N/A). | | | | |
| --- | --- | --- | --- | --- |
|  | **Leaf** | **Leaf** | **Leaf** | **Flower** |
| Samples (WT) | **WT2** | **WT3** | **WT4** | **WT2** |
| Technical replicate 1 | N/A | N/A | N/A | N/A |
| Technical replicate 2 | N/A | N/A | N/A | N/A |
| Technical replicate 3 | N/A | N/A | N/A | N/A |
| Technical replicate 4 | N/A | N/A | N/A | N/A |
| Sample (HC-Pro) | **HC-Pro1** | **HC-Pro2** | **HC-Pro3A** | **HC-Pro1** |
| Technical replicate 1 | 18.85 | 20.38 | 26.15 | 18.15 |
| Technical replicate 2 | 19.21 | 20.75 | 26.68 | 18.25 |
| Technical replicate 3 | 19.09 | 20.39 | 26.9 | 18.74 |
| Technical replicate 4 | 19.14 | 20.85 | 26.82 | 18.44 |
| Mean | 19.0725 | 20.5925 | 26.6375 | 18.395 |
| s.e. | 0.078142 | 0.12154 | 0.168739 | 0.129775 |

Three biological replicates of leaf samples (WT and HC-Pro) and one flower sample (WT and HC-Pro) are presented in the columns. Four technical replicates are also shown.
