# Supplementary material for: HC-Pro silencing suppressor significantly alters the gene expression profile in tobacco leaves and flowers
Source: BMC Plant Biol. 2011 Apr 20;11:68. doi: 10.1186/1471-2229-11-68 (PMC3111369; doi:10.1186/1471-2229-11-68)
Supplement: Additional file 6 — Supplemental Figure 6. A BOX-PLOT presentation of data based on Supplemental Tables 3 and 5 [file 1471-2229-11-68-S6.PDF]

Additional file 6. Quality control of the array results

WT/HC-Pro LEAF

Box-Plot

9959 elements Student's t-test (FDR p<0.1)

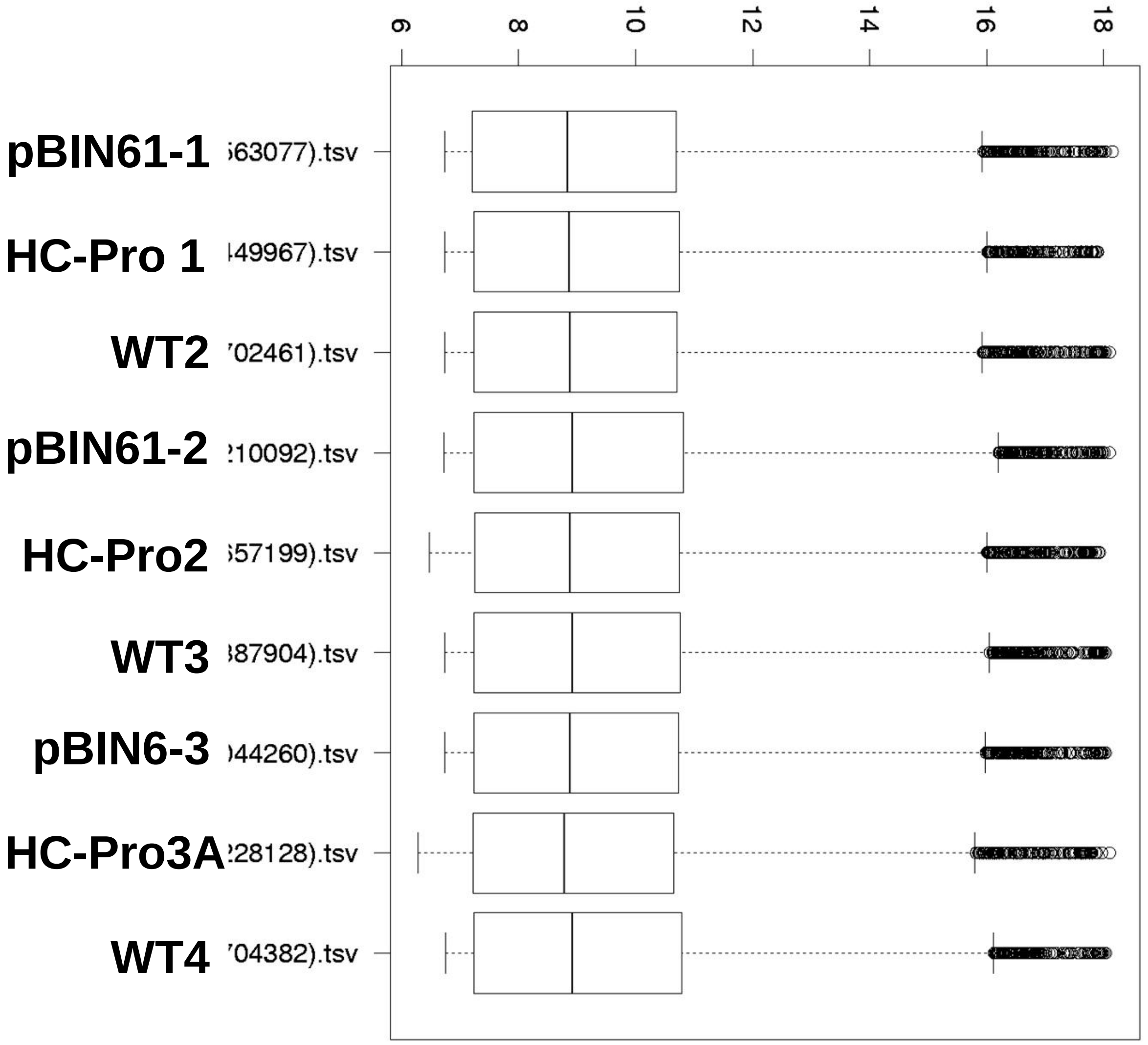

WT/HC-Pro LEAF

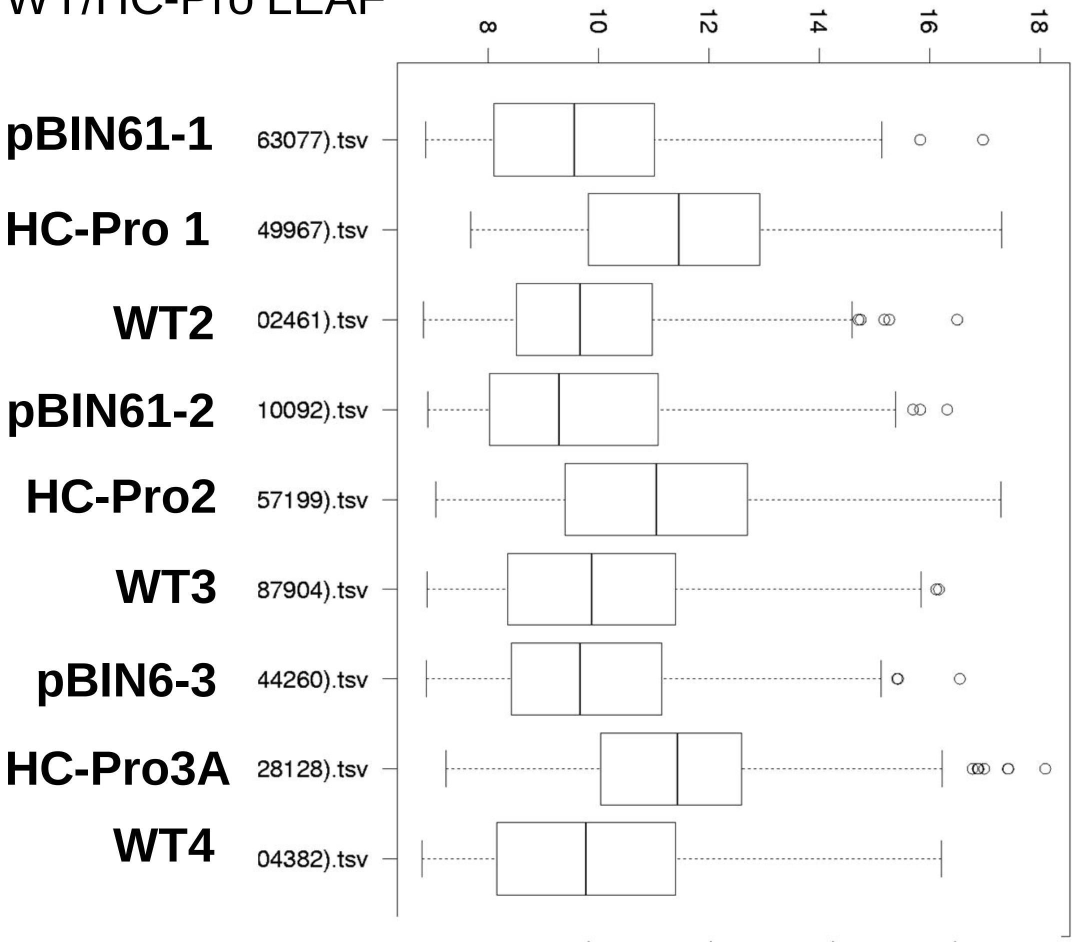

More than 2-times up regulated transcripts (FDR, $p<0.05$) 188 genes

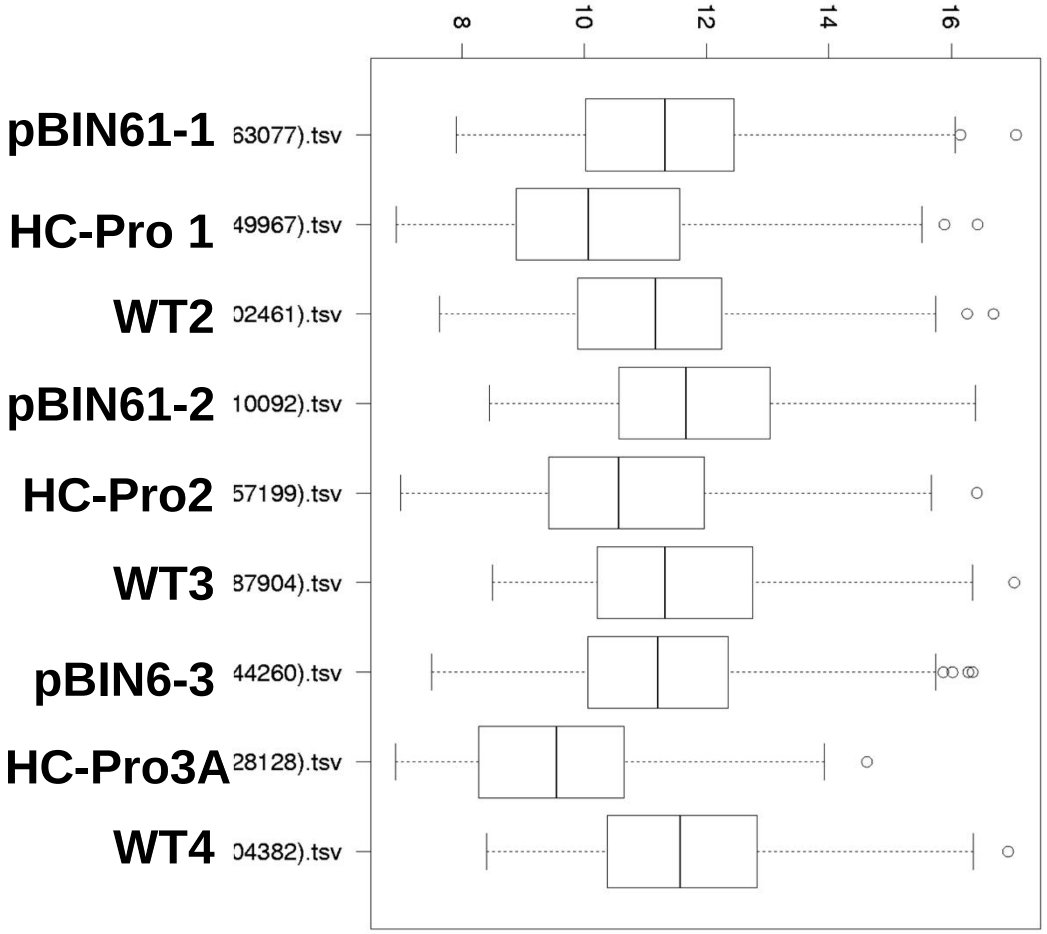

More than 2-times down regulated transcripts (FDR, $p<0.05$) 172 genes

WT/HC-Pro FLOWER

Box-Plot

11072 elements Student’s t-test (FDR $p<0.1$)

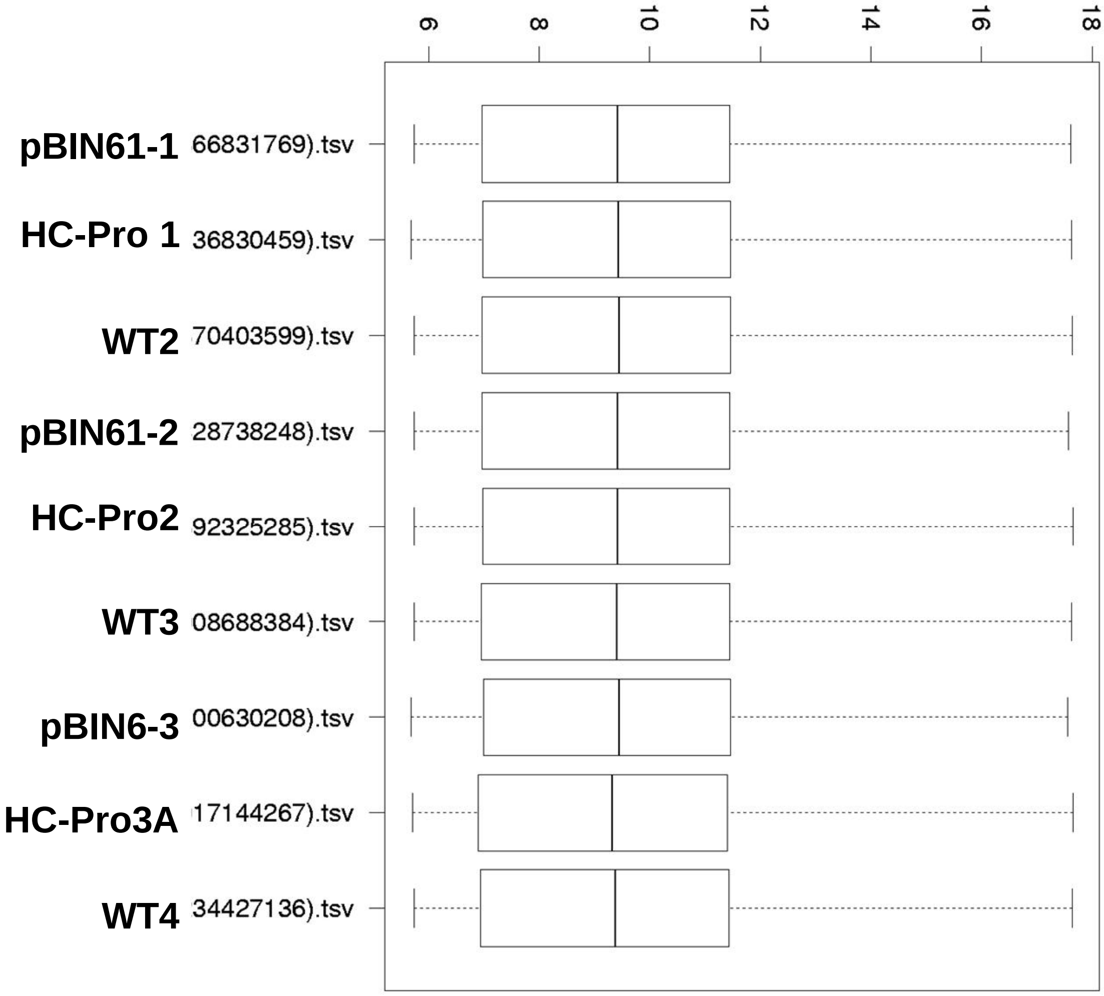

WT/HC-Pro FLOWER

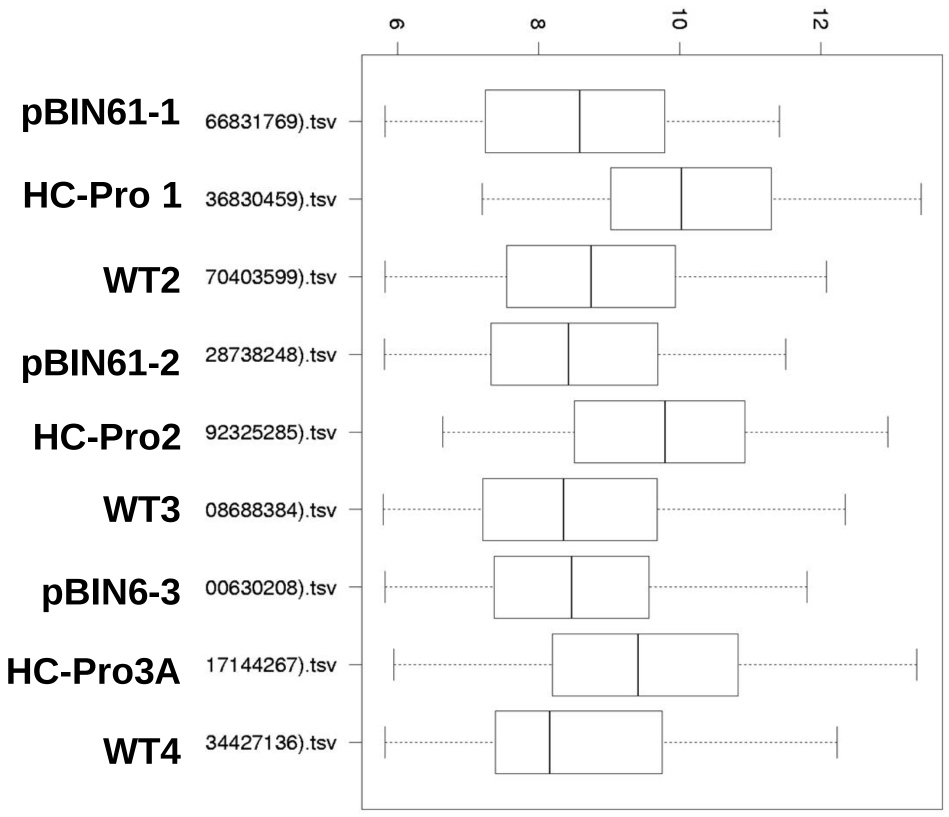

More than 2-times up-regulated transcripts (FDR<0.05) 71 genes

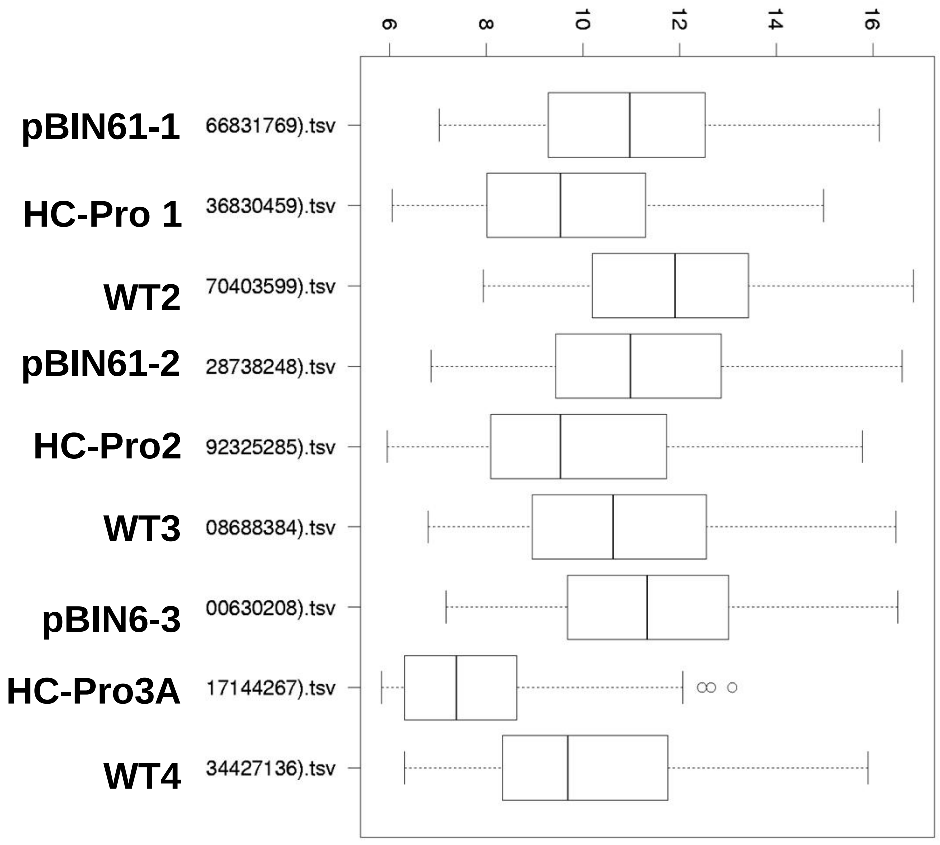

More than 2-times down-regulated transcripts (FDR<0.05) 125 genes
