# Supplementary material for: HC-Pro silencing suppressor significantly alters the gene expression profile in tobacco leaves and flowers
Source: BMC Plant Biol. 2011 Apr 20;11:68. doi: 10.1186/1471-2229-11-68 (PMC3111369; doi:10.1186/1471-2229-11-68)
Supplement: Additional file 7 — Method and results of starch quantification. [file 1471-2229-11-68-S7.DOC]

**Additional file 7.** Determination of starch content in the wild-type and HC-Pro expressing transgenic leaves

Tobacco leaf discs were cut from wild type and transgenic HC-Pro expressing plants, and weighed accurately (about 50 mg). Discs were frozen in liquid nitrogen and thawn in a plastic tube. Soluble sugars were first eluted using 400μl 80% Ethanol in 0.25 mM Hepes/KOH pH 7.5, 400μl 50% Ethanol 1 mM Hepes/KOH pH 7.5, and 200μl 80% Ethanol in 0.25 mM Hepes/KOH pH 7.5, each step for 30 min at 80^O^C. Total starch was determined using Megazyme total starch assay procedure (K-TSTA 04/2009) kit according to manufacturer’s recommendations. Leaf discs were air dried with nitrogen gas and grounded to powder after freezing with liquid nitrogen. After grinding, 200μl of 80% ethanol and 3 ml of solution 1 (thermo stabile α-amylase) was added. Samples were boiled in a kettle for 12 min mixing every 4 minutes. 100μl of amyloglucosidase (330U on starch) was added and incubated at 50^O^C for 30 min. Samples were centrifuged at 7000 rpm for 10 min, and 100μl of the clear supernatant was used for determination of starch after starch had been degraded to glucose. For measuring the amount of formed glucose, 3 ml of GOPOD-reagent was added. After 20 min incubation at 80^O^C for 30 min, the samples were measured in a spectrophotometer at 510nm against water control. 0.1 mg of D-glucose (1mg/ml) was used as a standard sample. Leaf samples consisted of four biological replicates, including two technical replicates of each.

| Leaf | Starch%/FW | Mean (AVG) | STD | SE |  | % starch/Control |
| --- | --- | --- | --- | --- | --- | --- |
| WT1 | 1.779587104 |  |  |  |  |  |
| WT2 | 1.74 |  |  |  |  |  |
| WT4 | 2.89 |  |  |  |  |  |
| WT6 | 2.4 | 2.2024 | 0.549 | 0.274533548 | n=4 | 100 |
| HC-Pro1 | 0.737 |  |  |  |  |  |
| HC-Pro2 | 0.360278638 |  |  |  |  |  |
| HC-Pro4 | 0.294868421 |  |  |  |  |  |
| HC-Pro6 | 0.827699443 | 0.555 | 0.267 | 0.133252213 | n=4 | 25.198 |
